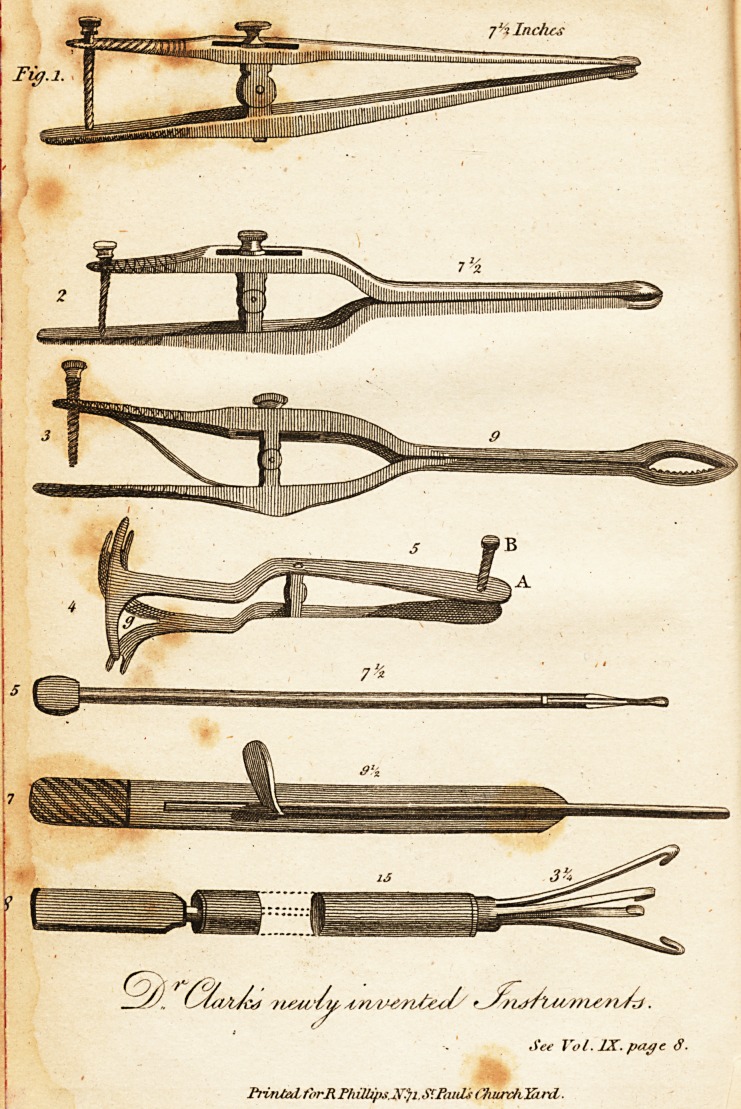# An Account of Surgical Instruments, Lately Invented

**Published:** 1803-01-01

**Authors:** Thomas Clark


### Medical and Physical Journal.

VOL. IX.] JANUARY 1, 1803. [NO. XLVII.

*Printed for R. PHILLIPS, by W. Thorne, Red Lion Court, Fleet Street, London.*

### *An Account of Surgical Instruments, lately invented by* THOMAS CLARK, *M. D.*

[ With Engravings. ]

IT appearing to me, that hitherto there was no surgical instrument well adapted to the enlargement of *gun-shot wounds* equally throughout their whole course, and particularly deep ones, I have invented an instrument which, in my opinion, will answer the intended purpose when wounds proceed in straight directions.

It consists of a straight director and a double edged knife, sharp on both sides near to its point, with a long ridge on its back, accommodated to the size of the groove of the director.

The mode of using this instrument is as follows: The director being cautiously introduced to the bottom of the wound, and placed as nearly as possible in its centre, it is then to be firmly retained in this situation by the left hand. The extremity of the ridge on the back part of the knife next to its point is to be introduced into the exterior extremity of the groove of the director by the right hand. This being done, let the knife be gradually passed along the director until it reaches the bottom of the wound. The knife and director may then either be withdrawn separately or both together. In some cases it may perhaps be proper to move the point of the knife a little from side to side at the bottom of the wound in order to enlarge it somewhat more near to the seat of the ball than elsewhere, with a view to render it more easy to lay hold of the ball.

As the groove of the director and the ridge of the knife are so constructed as effectually to prevent their being disengaged from one another, unless at the extremity of the director next its handle there is not the smallest risk of the parts of the instrument separating during the operation.

And as the edges of the knife are blunt, except near its point, it must of necessity be withdrawn from the wound precisely by the same passage through which it entered.

### *Observations on the Nature of the Forceps commonly used for extracting Balls from Gun-shot Wounds; together with an Account of several Pairs of Forceps.*

After fully considering the principles upon which forceps generally in use for the extraction of musquet-balls from wounds are formed; it occurred to me that they are liable to several objections which might be easily remedied.

In the first place, in extracting a ball with common forceps, if it is not situated between the blades near to their points, the points of the forceps, of necessity, will be at a greater distance from each other than the diameter of the ball, as the blades of the common forceps diverge from each other like radii of a circle. Hence, the farther a ball is placed from the points of the forceps, the greater will be the distance between them; consequently, it must be granted that great injury and inconvenience will occasionally happen in extracting balls with the common forceps, when they are situated at a considerable distance from their points.

Another and perhaps a more material disadvantage of the common forceps seems to be, that after they are in contact with the ball, although their points may be expanded to a distance equal to the ball's diameter, still it very often happens that while one of the blades might be passed on so as to be properly applied to the ball, that it is prevented from passing on, owing to the point of the other blade being immediately opposed by the ball. In such a case, I think it must be allowed that forceps constructed with blades capable of being moved easily backwards and forwards, so that one blade may be passed along, and be properly applied to the ball, while the other is prevented from being applied in a like manner, owing to its point being opposed by the anterior part of the ball, might possess considerable advantages over the common forceps. For it seems more than probable, that after one of the blades has been properly applied to the ball, that the other might also, after several attempts, be applied in a similar manner; as, surely, every one must grant that it is much easier to direct one blade of a pair of forceps at once, than to direct both blades at the same time.

With

With a view to obviate these objections, I have invented the forceps delineated fig. 1.

When the blades are parallel, their distance from each other is exactly equal to the diameter of a musquet ball; the intermediate part in which the hinge is, being precisely equal in length to the diameter of a musquet ball. The blades can be easily moved backwards and forwards, in such a manner that either may be easily made to project beyond the other for about half an inch, which distance I should imagine to be quite sufficient on most occasions. However, if half an inch is not considered as enough, forceps may very easily be constructed so as to admit of a much greater degree of motion. Therefore it must be allowed, that the being able to move the blades in the manner now described, will be often attended with considerable advantage. Lastly, as the blades of the forceps are only parallel when separated to a distance equal to the diameter of a musquet ball; and as their points, until the blades become parallel, approach nearer to each other than any other parts of the blades, it, in my opinion, must also be allowed, that the points of the forceps last mentioned, will not be so apt to meet with resistance from the soft parts, as the points of the common forceps would have been. The blades of the common forceps constantly diverge from each other as radii of a circle; but the blades of the forceps invented by me, do not diverge from one another in the least, until after they become parallel. And even after their points are separated to a much greater distance than the diameter of a musquet ball, they by no means diverge from each other so suddenly as the points of the common forceps. Hence, at all events, we must infer, that as the points of the forceps with moveable blades, until they become parallel, are constantly nearer to one another than any other part of the blades, that the part of the blades nearest to their points being at a greater distance from each other than the points themselves, will in a great measure prevent the softer parts from being opposed to the points of the blades, by the blades immediately behind their points, as it were, in a great measure removing the soft parts which otherwise would be opposed to them.

Upon the whole, therefore, I am decidedly of opinion that the forceps of my invention, with blades capable of being moved backwards and forwards, as already described, possess several very important advantages over those in common use.

It may be said that these forceps cannot be conveniently introduced into deep wounds, on account of the distance of the blades from each other near to the hinge. But if this distance is only equal to the diameter of a musquet ball, such an objection must be ill founded, as the whole wound necessarily will be distended equal to the ball's diameter, before it can be extracted. However, with a view to do away such an objection, I have had forceps bended, as represented in fig. 2. That part of the forceps near, to their point, intended for receiving the ball, remains in the same condition as if the blades had not been bended. Therefore, when a musquet ball is any where included between these parts, they will be parallel; consequently, it is of no importance whether the ball is placed near to the point of the forceps or a little farther back.

As at first sight, perhaps, the reasoning already advanced concerning these newly-invented forceps, may not appear altogether just to some, I have had a pair made pretty much on the same principles with the common forceps, but with this difference, that the blades may be easily moved backwards and forwards. Vide fig. 3.

Before quitting this subject entirely, I think proper to mention, that I am of opinion, that forceps for extracting stones from the urinary bladder, constructed on principles nearly similar to these lately mentioned, might be preferable to those commonly used. In this case, there would be no occasion for blades being capable of being moved backwards and forwards, &c.

### *A Description of an Instrument for removing Strictures of the Urethra.*

Strictures of the urethra being often productive not only of much real distress to those afflicted with them, but more frequently having been the occasion of death itself, has induced me to think seriously on the subject. It affords me infinite pleasure to say, that I have been fortunate enough to contrive a very simple instrument, which I should hope will prove highly beneficial in most cases, and will, in a great measure, supercede the hazardous application of caustic, or the introduction of sharp-pointed instruments.

The seat of the stricture being ascertained, a silver canula, including a round large blunt pointed probe, fitting exactly, and projecting a little beyond the canula, in order to prevent the urethra being injured by the canula, is to be be introduced into the urethra, and passed on until it reaches the stricture. The blunt probe is now to be withdrawn; the canula in the mean time being firmly retained in its situation. Then let the instrument, represented in fig. 5, be introduced into, and passed along the canula, until it reaches the stricture. It will now be necessary to endeavour to pass the silver probe attached to the point of the cutting instrument, through the stricture. As soon as this is effected, the cutting instrument must be pushed forwards, with a view to divide the stricture, which in most instances, I should suppose, will be easily done. The cutting instrument being withdrawn, a bougie ought to be introduced, either through the canula or after it has been removed, in order to ascertain, beyond the possibility of doubt, whether the stricture has been completely divided. If it has not been completely divided, it may be again proper to use the instrument, and thus effectually remove the disease. It seems almost unnecessary to say, that bougies ought to be introduced from time to time until the divided parts become perfectly sound.

The steel probe, with the lancet point, should fill the canula completely, and the breadth of the lancet should be somewhat less than the diameter of the steel probe. By these means, the silver probe will be directed precisely in the centre of the urethra, while, at the same time, the edges of the knife will not be blunted in passing through the canula. With regard to the utility of the silver probe, fixed to the point of the lancet, it seems so evident, that it appears almost unnecessary to say any thing concerning it. If the probe passes through the stricture, it must inevitably happen, that when the probe is pushed further in, that the knife will follow it, and consequently divide the stricture. That this mode of removing strictures is far preferable to any of the methods commonly practised, I think cannot admit of a single doubt.

The removing of strictures by means of bougies, at best, is a tedious and troublesome process; and, with regard to the use of caustic, or sharp-pointed instruments, they are not only tedious and uncertain remedies, but may be productive of the most dangerous consequences by destroying or puncturing the sides of the urethra, and thereby forming communications between the urethra and its cellular substance. Hence profuse hæmorrhages may take place, or urine may be affused into the cellular substance, and give rise to very violent inflammation, and its dreadful consequences. On the contrary, when this cutting instrument ment is used, there is not the least danger of the sides of the urethra being materially injured, as the silver probe must of necessity be lodged in the centre of the urethra, and the cutting part of the instrument will consequently follow the same course.

The instrument now described is evidently only calculated to remove strictures within the reach of straight instruments. However, I am of opinion, that an instrument upon the same principles may be easily constructed, so as to remove strictures in whatever part of the urethra they may be situated. The cutting part of the instrument, with the silver probe attached to it, might be easily fixed to a pliable piece of whale-bone or to a bougie. A canula of elastic gum being introduced into the urethra, as far as the stricture, an instrument, such as now mentioned, certainly could be passed through the canula, and used in a similar manner with the straight cutting instrument already described. In this way, I am of opinion that strictures, even at the neck of the bladder, might be removed.

### *An Account of an Instrument, &c. for removing Substances, such as Pins, Pieces of Money, &c. from the Œsophagus.*

No branch of Surgery appears to me more defective than that relating to the removal of substances lodged in the œsophagus, such as pins, pieces of money, bones, &c. I lately directed my attention to that subject; and, I believe I have been fortunate enough to invent an instrument which, on many occasions, will prove of infinite service.

It consists of a tube of elastic gum, and a long piece of whale-bone, to which four elastic steel hooks are fixed. Vide fig. 8.

The tube, including the whale-bone, with its appendages, the extremities of the hooks being just at the further extremity of the tube, is to be introduced into the œsophagus and pushed gradually downwards until it reaches the substance that may happen to be lodged there. Let the tube now be withdrawn about two inches, and kept firmly in its situation by the left hand; then let the whale bone be forced gradually downwards, by the right hand; for about three inches. As soon as the points of the elastic hooks pass a little beyond the tube, they will begin to gradually expand themselves; and, by the time they have passed the extremity of the tube for about two inches, they will be expanded to such a degree as to distend the œsophagus completely.

pletely. This being the case, if they are pushed downwards for another inch, or thereabouts, the presumption is, that they will have passed beyond the substance to be extracted, and owing to their number, nature and situation, it is more than probable, that some of the hooks will have passed on each side of it. The whale-bone is now to be firmly retained in this situation, and the tube in the mean time to be gradually pushed along the whalebone for about two inches. In other words, the tube is to be passed downwards until it again reaches the substance to be extracted. This being accomplished, it will be proper to keep the tube in its present situation, and attempt to withdraw the whale-bone. If the substance to be extracted is small, and capable of being bended easily, it may be withdrawn through the tube. Pins, or bits of wire, might be removed in this manner. Or, if the instrument has not laid hold of the substance, it will of course be readily withdrawn; however, I should hope that this will seldom happen. Again, if the substance to be extracted cannot be made to pass through the tube, the making an effort as it were to withdraw the whale-bone will be necessary, in order to fix the substance properly in the hooks. This being done, the tube, along with the whale-bone, must be carefully and very gradually removed; and I believe I may venture to say, that substances, after being secured in the manner now described, will be very seldom disengaged from the instrument until they are completely removed from the œsophagus.

Upon the whole, I do not hesitate to give it as my opinion, that instruments constructed on principles similar to those of that now described, will be found preferable to any instrument heretofore used for extracting substances from the œsophagus.

An instrument with only two or three hooks, I should imagine, would answer the desired purpose in most instances. But if a greater number of hooks than four should at any time be deemed requisite, it will be a very easy matter to make an instrument with six or eight hooks.

### *An Account of a Tourniquet, &c. for the Tongue.*

When it becomes necessary from cancerous sores or other causes, to extirpate a portion of the tongue, the profuse hæmorrhagies which almost constantly ensue, are often the source of great embarassment to the operators, and not unfrequently endanger the lives of the patient. Nay, it seems to me extremely probable that many lives have been lost from from the want of an instrument capable of commanding the circulation in the tongue. In some instances, the dread of profuse hæmorrhagies may have deterred practitioners from operating on the tongue, when, from the other circumstances, an operation might have been extremely proper. In other instances, it is very probable that profuse hæmorrhagies, in consequence of operations performed on the tongue, may have ultimately occasioned death.

From these very important considerations, I have been induced to exert my faculties to the utmost, with a view to invent an instrument which might at pleasure command the circulation in the tongue. Notwithstanding the instrument which I have invented is only calculated to compress the tongue as far back as the frænum-linguæ extends, still however an instrument capable of producing this effect, is on many occasions a very desirable object. By means of it, operations may be performed nearly as far back as the frænum extends, with the utmost safety; that is, so far as regards the restraining of hæmorrhagies.

### Description of the Instrument, Fig. 4.

The blades or divisions of the instrument are separated from each other by depressing the extremity of the upper one at (A). This being done, the under part or division of the instrument is to be placed under the tongue, and the anterior part of the frænum being received into the slit at (S), the instrument should be pushed as near to the root of the tongue as possible. The extremity of the upper part of the instrument next to the root of the tongue is now to be made to approach the extremity of the part of the instrument under the tongue; to such a degree as to stop the circulation in the anterior part of the tongue. This effect is very easily produced by separating the extremities of the blades of the instrument without the mouth by means of the screw nail (B) passing through the upper one.

Thus the circulation of blood in the anterior parts of the tongue may be at pleasure effectually stopped, and consequently the danger attending operations there, will be rendered much less than otherwise would have been.

## Figures and Tables

**Fig. 1. 2 3 4 5 7 8 f1:**